# Localization of recurrent lesions following ileocolic resection for Crohn’s disease

**DOI:** 10.1186/s12893-020-00980-9

**Published:** 2021-03-20

**Authors:** Hiroki Ikeuchi, Motoi Uchino, Toshihiro Bando, Yuki Horio, Ryuichi Kuwahara, Tomohiro Minagawa, Yoshiko Goto, Kurando Kusunoki, Masataka Ikeda, Naohito Beppu, Yoshio Takesue

**Affiliations:** 1 Department of Gastroenterological Surgery, Hyogo College of Medicine, 1-1 Mukogawa-cho, Nishinomiya, Hyogo 663-8501 Japan; 2 Division of Infection Control and Prevention, Hyogo College of Medicine, 1-1 Mukogawa-cho, Nishinomiya, Hyogo 663-8501 Japan

**Keywords:** Crohn’s disease, Ileocolic resection, Surgical recurrence, Recurrence site

## Abstract

**Background:**

Crohn’s disease (CD) recurrence can occur not only at the site of anastomosis but also elsewhere in the bowel following an ileocolic resection (ICR) procedure. The aims of the present study were to assess long-term outcomes of a primary ICR procedure for CD in consecutive patients and examine the location of the reoperation causative lesion.

**Methods:**

We examined cases of surgery with ICR initially performed at our institution. Those with simultaneous multiple bowel resection or bowel resection with strictureplasty were excluded.

**Results:**

A total of 169 patients who underwent ICR due to CD were enrolled. The median follow-up period was 12.6 years (range 4–27 years). A reoperation was needed in 45 (26.6%), of whom 14 had lesions causative of the reoperation at other than the anastomotic site. The most common causative lesion location was in the colon rather than the oral side of the small intestine. Furthermore, we investigated the relationship between presence of residual lesions following the initial surgery and lesions causative of reoperation. In the group without residual disease (n = 31), 29.0% (n = 9) had non-anastomotic lesions involved in indications for reoperation, while that was 35.7% (n = 5) in the group with residual disease (n = 14).

**Conclusions:**

Anastomotic site lesion is not the only causative factor for reoperation following ICR. Regular examinations and applicable treatment with awareness that the cause of reoperation is not limited to the site of anastomosis are important in these cases.

## Background

Crohn’s disease (CD) is a chronic inflammatory disease that can potentially involve the entire gastrointestinal tract. Long-standing inflammation causes irreversible damage to the bowel wall, resulting in either fibrotic strictures or penetrating disease. Conservative medical treatment is considered to be the principal therapeutic approach to treat patients with CD, though up to 80% will ultimately require surgery [1]. Moreover, a repeat operation for recurrent CD has been estimated to be required in up to 40% of surgically treated cases after 10 years [2].

The anastomotic site following CD surgery is where recurrence is most likely to occur. Recently, there has been an increasing number of reports showing that results of treatment were improved by providing step-up therapy based on endoscopic findings of the site of anastomosis. On the other hand, in some cases of reoperation, the lesion causing the reoperation is not located at the anastomotic site of the initial operation. It is important to note that there may be gaps between endoscopic, clinical, and surgical recurrence in CD cases. Most published studies have not addressed surgical recurrence as the end-point and enrolled a low number of patients, thus surgical recurrence has not been well addressed.

The aim of the present study was to assess long-term outcomes of a primary ileocolic resection (ICR) for CD in consecutive patients and clarify the location of the causative lesion related to the reoperation.

## Methods

### Patients and data collection

The authors developed, maintained, and upgraded a database containing clinical findings of all CD patients who underwent an operative procedure at our institution, with data entered on a prospective basis and updated with each new instance of recurrence. This database was used retrospectively to investigate CD recurrence sites for the present study.

From January 1990 to December 2015, 1143 patients with CD underwent surgery at the Department of Gastroenterological Surgery, Hyogo College of Medicine. Of those, cases of initial surgery with ICR were selected. Patients who underwent simultaneous multiple bowel resection and bowel resection with strictureplasty procedures were excluded. Altogether, 169 patients who underwent ICR due to CD were included in the study. The median follow-up period was 12.6 years (range 4–27 years).

All reoperation procedures were explained by a physician specializing in inflammatory bowel disease, with the final decision determined based on discussions that included the surgeon and patient. All surgeries were performed by colorectal surgeons experienced in surgical management of CD. A hand-sewn end-to-end ileocolonic anastomosis was constructed as a two-layer anastomosis using a continuous inner layer reinforced with interrupted sero-muscular sutures. Postoperative prophylaxis with immunomodulators and/or biologic agents or other treatments were started 2–4 weeks after surgery at the discretion of the attending gastroenterologist.

### Definitions

**Fig. 1:**
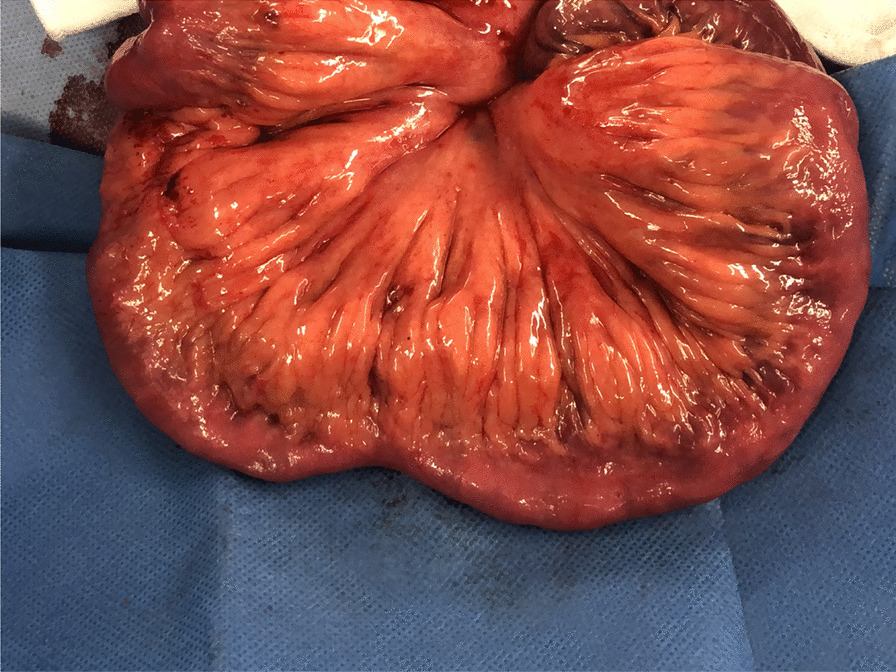
Small bowel lesions not targeted for resection. Representative case. The preoperative examination showed no obstruction, while intraoperative findings revealed CD lesions with creeping fat. Such cases were defined as having residual disease

CD was confirmed by a histological examination of the resected specimen. Surgical recurrence was defined as the necessity of a reoperation because of recurrent CD during the observation period. The causative lesion responsible for the reoperation was confirmed by endoscopy, contrast examination, and CT findings. Lesions considered responsible were classified as anastomotic, non-anastomotic, or both. When the preoperative examination results showed no obstruction but intraoperative findings revealed CD lesions with creeping fat, that case was defined as residual disease (Fig. 1).

### Statistical analyses

Descriptive statistics are reported as median values (range) and frequency (percent) for categorical variables. Survival data are presented as Kaplan–Meier curves and described by 5-year probability for surgical recurrence survival. All statistical analyses were performed using JMP version 11 (SAS Institute Inc., Cary, NC, USA).

### Ethical considerations

The study protocol was approved by the Ethical Committee of Hyogo College of Medicine (number 3539).

## Results

### Patient characteristics

Of the 169 patients enrolled, 117 (69.2%) were male and 52 (30.8%) were female. Mean age at onset of CD was 22 years (11–64) and mean age at the initial operation was 30 years (14–76). Patient demographics are presented in Table 1.

**Table 1:** Patient characteristics

Gender (male/female)	117/52
Age at onset of Crohn’s disease, years (range)	22 (11–64)
Age at initial operation, years, (range)	30 (14–76)
Disease location at surgery, ileum/colon/both	95/7/67

### Surgical indications

Indications for the initial procedure for all cases are shown in Table 2, with stenosis found to be most common. Five patients who received the initial surgery for perforation underwent an emergency procedure, while the others had elective surgery.

**Table 2:** Indication for initial operation (n = 169)

Stenosis	88 (52.1%)
Abscess	38 (22.5%)
Fistula	30 (17.8%)
Bleeding	8 (4.7%)
Perforation	5 (3.0%)

### Postoperative complications

Postoperative complications were noted in 10 patients (5.9%), with details shown in Table 3. Wound infection and intra-abdominal abscess were the most common following the initial operation, though no cases required a reoperation due to a postoperative complication.

**Table 3:** Postoperative complications after initial operation

Wound infection	3 (1.8)
Intra-abdominal abscess	3 (1.8)
Anastomotic leakage (minor)	1 (0.6)
Anastomotic bleeding	1 (0.6)
Sepsis	1 (0.6)
Sub-ileus	1 (0.6)

### Indications for reoperation

A reoperation for recurrence was needed in 45 (26.6%) patients, which was manifested as stenosis in 28 (62.2%), fistula in 7 (15.6%), abscess formation in 6 (13.3%), and exacerbation of an anorectal lesion in 4 (8.9%) (Table 4). All four cases in which exacerbation of rectal-anal lesions were causative of the reoperation were associated with stenosis of the rectum or anal canal. All re-surgery cases were elective procedures.

**Table 4:** Indication for re-operation (n = 45)

Stenosis	28 (62.2%)
Fistula	7 (15.6%)
Abscess	6 (13.3%)
Exacerbation of anorectal lesion	4 (8.9%)

### Localization of lesions causing reoperation

Forty-five patients required a reoperation due to recurrence of CD. Table 5 shows details of the causative lesion. Fourteen of these patients had causative lesions in other than the anastomotic site.

**Table 5:** Localization of regions causing reoperation (n = 45)

Anastomotic region	31 (68.9%)
Site other than anastomotic region	10 (22.2%)
Both regions	4 (8.9%)

### Localization of causative lesions other than anastomosis

Table 6 shows localization of lesions in the 14 cases in which lesion location other than the anastomotic region caused the reoperation. There were some overlaps. The most common was a lesion in the colon rather than the oral side small intestine. Intraoperative findings obtained at the initial surgical procedure in these reoperation cases showed no residual lesion in the colon.

**Table 6:** Localization of causative lesions other than anastomosis

Ileum	3
Colon	7
Rectum	2
Anal lesion	5

### Presence of residual lesion and localization of lesion causing reoperation

Relationships between presence or absence of residual lesions in the proximal small intestine at the time of the initial surgery and localization of lesions responsible for the reoperation are shown in Table 7. Non-anastomotic lesions were involved in the surgical indications for reoperation in 9 (29.0%) of 31 patients without residual disease and 5 (35.7%) of 14 with residual disease.

**Table 7:** Presence of residual lesions and localization causing reoperation

	Residual lesion (−)	Residual lesion (+)
	(n = 31)	(n = 14)
Anastomotic region	22 (71.0%)	9 (64.3%)
Site other than anastomotic region	7 (22.6%)	3 (21.4%)
Both regions	2 (6.5%)	2 (14.3%)

(−) Macroscopic Crohn’s lesions in residual intestinal tract

(+) Macroscopic Crohn's lesions in residual intestinal tract

### Cumulative 5-year reoperation rate

**Fig. 2:**
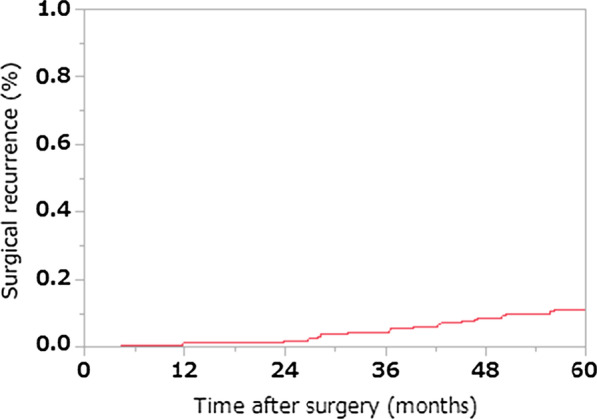
Cumulative 5-year reoperation rate. The cumulative reoperation rate after 5 years in the present cohort was 11.3%

The cumulative reoperation rate is presented in Fig. 2. That after 5 years was 11.3% in the present cohort.

## Discussion

The present findings revealed a difference between endoscopic recurrence in the anastomotic site and the causative lesion related to the reoperation. Among other studies presented thus far, Aaltonen et al. reported that the majority of cases of surgical recurrence (71.4%) were because of a lesion located in the proximal portion of the site of anastomosis and required a new ileocolic resection [3], while other studies also concluded that surgical recurrence after an ileocolic resection is more common in the anastomotic site [4, 5]. Furthermore, the reoperation rate after stoma construction is high in CD patients. In a recent report, Koriche noted a surgical recurrence rate of 38% with a median follow-up of 2.4 years [6]. Based on these results, step-up therapy based on endoscopic findings of the anastomosis site or oral lesions of the stoma is generally given to affected patients.

In a study performed approximately 30 years ago, Rutgeerts et al. showed that the severity of mucosal lesions was correlated with likeliness to develop clinical recurrence [7]. Thereafter, the so-called Rutgeerts endoscopic index has been used to define the primary endpoint in most randomized controlled trials conducted in the recent decade to evaluate preventive strategies for postoperative recurrence (POR) as a surrogate for clinical recurrence. Although POR is usually seen in the neoterminal ileum after ICR, some patients may develop mucosal lesions in another intestinal area. This is not considered when determining Rutgeerts score.

On the other hand, Fichera et al. noted that even though it is commonly believed that Crohn’s recurrence is always located at the site of a previous intestinal anastomosis, that concept is probably not true and not mindful of the pan-intestinal nature of the disease [8]. In that study, the authors reported that while the site of original operative intervention is the most common for recurrence, as many as one-third of recurrence cases occur in a location separate from that. In the present study as well, 14 (31.1%) cases had lesions away from the anastomotic site that were causative of the reoperation. In addition, abdominal findings obtained at the first operation, even in cases with no macroscopic residual lesions, showed that 9 (29.0%) of 31 of the lesions causative of the need for a reoperation were located in other than the anastomotic region. In the future, an intraoperative lesion search will also require technological progress without relying on the experience and inspection ability of the attending surgeon. In this regard, Celentano et al. reported the usefulness of an intraoperative ultrasound examination of the small bowel in Crohn’s disease patients [9].

Recently, in order to reduce the number of reoperations caused by anastomosis, improvements in associated treatment methods have been made, with endoscopic balloon dilation of the stenosis site commonly performed. To prevent surgical recurrence at the anastomotic site, Kono et al. developed a new antimesenteric functional end-to-end anastomosis technique in 2003 termed Kono-S anastomosis [10]. With this technique, the supporting column prevents distortion and keeps the intestinal tract in the anastomosis region straight, which also makes endoscopic examination and balloon dilatation following surgery easier to perform. Shimada et al. reported that the 5-year surgery-free survival rate at the site of anastomosis in patients who underwent Kono-S anastomosis (95%) was significantly higher as compared to those who received an end-to-end anastomosis procedure (81.3%; p < 0.001) [11]. Furthermore, in the first randomized clinical trial to compare Kono-S and conventional side-to-side anastomosis procedures in CD patients, Luglio et al. reported results demonstrating a significant reduction in postoperative endoscopic and clinical recurrence rates for patients who underwent Kono-S anastomosis [12].

Reports of the usefulness of balloon dilatation for stenosis in CD cases are increasing. Shivashankar noted that endoscopic stricture dilatation in CD patients was safe and effective, and that the most common stricture location was ileocolonic anastomosis [13]. Ding as well reported that endoscopic balloon dilatation of an anastomotic stricture in Crohn’s cases is safe and effective over the long term [14]. Based on these results, it is considered possible that lesions in locations other than the anastomotic site will cause a relatively high rate of reoperation.

Alternative non-invasive imaging techniques used to assess postoperative recurrence have been evaluated in recent years [15–17]. Wireless capsule endoscopy has potential advantages over ileocolonoscopy, as it is more comfortable and better tolerated by patients, does not require sedation, and is less influenced by technical limitations. Wireless capsule endoscopy was shown able to detect mucosal lesions one year after surgery in the upper segment of the gastrointestinal tract, outside the area of visualization by ileocolonoscopy, in 60% to 70% of examined patients. Furthermore, in recent years, the number of reports showing the usefulness of minimally invasive magnetic resonance (MR) enterography for evaluation of lesions of CD has been increasing [18, 19].

In future examinations, not only the anastomotic site, but all remaining intestinal tracts should be checked regularly using a minimally invasive method such as capsule endoscopy or MR enterography. In patients with a newly diagnosed lesion, it may be possible to avoid a reoperation by performing step-up therapy.

The present study has some limitations. First, this was a retrospective review of a prospective database of cases experienced at a single center, thus all limitations inherent to a retrospective study apply. Second, the gastroenterologist in charge of the follow-up examinations assessed the need for therapeutic modification on an individual patient basis, without reference to a predefined protocol.

## Conclusion

It is important to recognize that there may be a discrepancy between endoscopic or clinical and surgical recurrence in CD patients. In such cases, it is expected that the incidence of reoperations due to an anastomotic stricture will be reduced by improving the anastomosis method and advancing balloon dilatation. We consider that greater attention should be given to relapse in the remaining intestinal tract, thus recommend regular examinations and treatments with due recognition that the cause of reoperation may not be limited to the site of anastomosis.

## Data Availability

The data sets supporting the conclusions stated in the text are included within the article. Data are available from the corresponding author upon reasonable request.

## Abbreviations

CD: Crohn’s disease
ICR: Ileocolic resection
